# The levels of proinflammatory cytokines in blood serum of patients with juvenile idiopathic arthritis

**DOI:** 10.1186/1546-0096-12-S1-P183

**Published:** 2014-09-17

**Authors:** Faina Rokhlina, Gennadiy Novik

**Affiliations:** 1 SPbGPMU, St.Petersburg, Russian Federation

## Introduction

IL-1, IL-6 and TNF-alpha is a typical example of a multifunctional cytokine that is involved in the immune response, hemopoiesis and inflammation. In 1999 published data on the levels of cytokines in patients with JRA. It is noted that the level of IL-6 higher in patients with systemic variant of the disease in the active phase. This study confirms that levels of IL-6 and TNF-alpha can be markers of disease activity.

## Objectives

The aim of our study was to determine the relationship between the level of cytokines and disease activity among children with JIA receiving the basic therapy with methotrexate.

## Methods

Determination of the level of IL-1β, IL-6 and TNF-alpha serum was collected by means of enzyme-linked immunosorbent assay.

## Results

We have revealed significantly higher levels of IL-6 in groups of patients with oligoarticular (52.99±12.03) and system arthritis (68.76±14.7), compared with the level of IL-6 in patients with enthesitrelated arthritis (15.53±4.63) (P<0.05).

When comparing the levels of cytokines in patients with "Active" and "Inactive" disease depending on the type of arthritis, identified the following regularities in patients with systemic JIA in the stage inactive disease, the level of IL-6 (83.36±26.29) significantly (P<0.05) higher than these patients with polyarticular (14.65±6.64) and enthesitrelated arthritis (4.86±1.44). The level IL-6 significantly higher in patients with systemic arthritis in "active" phase (61.46±18.39) compared with the same patients with enthesitrelated JIA (16.86±5.13).

Noteworthy detection of statistically significant higher level TNFα in patients receiving biological therapy, mainly TNF inhibitors. Apparently, the continuing high level of TNF, despite the use of inhibitors, characterizes this group of patients, as the most severe.

In the group of patients not treated with biological therapy level TNFα was significantly lower than in the group of children treated with this type of therapy. The main biological drugs, which were given to children with Jia were either direct blockers TNFα (infliximab, etanercept) or affecting the synthesis of Pro-inflammatory cytokines (tocilizumab, adalimumab).

## Conclusion

It was natural to assume that in a group of children receiving anticytokine drugs the level of TNF-alpha will be lower. However, in our study we have received significantly higher level of TNF-alpha, which indicates a more severe disease, and on the other hand possible resistance to selected for treatment of biological drugs in these patients.

## Disclosure of interest

None declared.

